# A cross-sectional and 6-year follow-up study of associations between leisure time physical activity and vertebral fracture in adults

**DOI:** 10.1186/s12891-019-2821-8

**Published:** 2019-09-17

**Authors:** Saija Mikkilä, Giovanna Calogiuri, Nina Emaus, Bente Morseth

**Affiliations:** 1 School of sport sciences, Faculty of Health Sciences, UiT The Arctic University of Norway, Tromsø, Norway; 2 Department of Community Medicine, UiT The Arctic University of Norway, Tromsø, Norway; 3 Department of Dental Care and Public Health, Inland Norway University of Applied Sciences (INN), Elverum, Norway; 4 Department of Health and Care Sciences, UiT The Arctic University of Norway, Tromsø, Norway

**Keywords:** Epidemiology, Osteoporosis, Exercise

## Abstract

**Introduction:**

Vertebral fractures are common osteoporotic fractures, affecting 2–46% of the population, causing morbidity and increased risk of mortality. Physical activity has beneficial effects for bone health, including increased bone mineral density and reduced hip fractures. However, evidence concerning prevention of vertebral fractures is scarce. Therefore, the aim of this study was to investigate the association between leisure time physical activity and vertebral fracture risk.

**Methods:**

The data were retrieved from the 2001 and 2007–2008 surveys of the Tromsø Study, a longitudinal population study in Norway. A total of 1904 participants (1030 women and 874 men, age 38–87 yr and 40–87 yr respectively) were included in the cross-sectional analysis (2007–2008). Prospective follow-up data (2001 to 2007) on physical activity were available for 1131 participants (636 women and 495 men, age 32–69 yr and 33–69 yr respectively). Physical activity was assessed by a questionnaire and vertebral fracture by lateral vertebral fracture assessment from dual-energy x-ray absorptiometry scans. Logistic regression was used to examine associations between physical activity and vertebral fracture.

**Results:**

After controlling for confounders (age, height, weight, smoking, osteoporosis, osteoporosis medication, left hip total bone mineral density, and use of hormones in women only), no cross-sectional associations between physical activity levels and vertebral fracture were observed, OR 1.13 (95% CI: 0.59–2.13), for moderately active women and 1.44 (0.61–3.42) for highly active women, compared with sedentary women. In men, the respective ORs were 1.74 (95% CI: 0.91–3.35) and 1.64 (0.78–3.41). In the prospective analyses, OR for vertebral fracture in women with reduced physical activity was 0.81 (95% CI: 0.18–3.62), 1.24 (95% CI: 0.29–5.26) for increased physical activity and 1.54 (95% CI: 0.43–5.50) for active unchanged physical activity pattern, compared with sedentary unchanged physical activity. In men, the respective ORs were 2.05 (95% CI: 0.57–7.42), 2.23 (95% CI: 0.63–7.87), and 1.81 (95% CI: 0.54–6.02). Subanalyses of women and men ≥50 yr showed similar results.

**Conclusions:**

Our findings suggest that physical activity does not play a major role in preventing vertebral fractures in Norwegian adults. Future studies may benefit from data on incident vertebral fracture, and objectively measured physical activity.

## Introduction

Vertebral fractures are common osteoporotic fractures, leading to reduced quality of life [1], morbidity and increased risk of mortality [2]. Moreover, having a vertebral fracture increases the risk of new fractures both in vertebra and in other locations [3]. A recent review on world wide prevalence of vertebral fractures showed variation by gender, ethnicity and geographical location from 3.2% (47–95 yr) to 37% (≥ 65 yr) in Japanese men and from 1.9% (45–69 yr) in British women to 46% (≥ 50 yr) in Moroccan women. However, different research methods and quality of studies hampers comparison of findings [4]. In Tromsø, northern Norway, the vertebral fracture prevalence has been estimated to 11.8% in women and 13.8% in men (38–87 yr), and as high as 19.2 and 20.3%, respectively, in the age group ≥70 yr. The observed prevalence is higher in men than in women [5].

In addition to the individual burden, osteoporotic fractures result in substantial economic costs for society. The economic burden of osteoporotic fractures in EU was estimated to €37 billion in 2010 of which the proportion of the vertebral fractures was 5% [6]. By preventing, rather that treating, the overall burden of vertebral fractures could be considerably reduced.

Physical activity has shown to have a beneficial effect for bone health and fracture risk [7]. However, whether physical activity can prevent vertebral fractures is scarcely elucidated [8] and especially longitudinal studies are needed on this topic. In addition, the majority of the studies have focused on the effect of the physical activity in rehabilitation of the vertebral fractures [9–11]. Therefore, the aim of this prospective study was to investigate the association between leisure time physical activity and osteoporotic vertebral fracture, as well as associations between changes in leisure time physical activity and the risk of vertebral fracture after a 6-year follow up in women and men who participated in the population-based Tromsø Study.

## Methods

### Design and subjects

The Tromsø Study is a longitudinal population-based study carried out in Tromsø, northern Norway [12]. It was initiated in 1974 with the first survey focusing mainly on studying cardiovascular disease in men. The study was later expanded to investigate a number of other health conditions and diseases e.g. physical activity and osteoporosis [12] in both men and women, and continued with six follow-up surveys in 1979–1980, 1986–1987, 1994–1995, 2001, 2007–2008 and in 2015–2016. The Tromsø Study is described more detailed in the Cohort Profile [12].

To each survey, Tromsø municipality residents were invited to participate and invitation letters were sent out based on the official population registry. Since 1994–95, all participants have signed a written informed consent declaration prior to inclusion. Further, The Tromsø Study has been approved by the Data Inspectorate of Norway and Regional Committee of Medical and Health Research Ethics, North Norway.

Data for this study is retrieved from the fifth and the sixth surveys of the Tromsø Study, which were carried out in 2001 and 2007–2008, respectively. Firstly, we performed a cross-sectional analysis by using the 2007–2008 data which included 1904 participants (1030 women and 874 men) aged 38–87 yr and 40–87 yr, respectively. Secondly, we performed a prospective analysis by using valid prospective data on changes in physical activity from the 2001 to 2007–2008. For the prospective analyses, participants with missing data for all the main variables and confounders in either 2001 or 2007–08 were excluded. This resulted in a prospective sample of 1131 participants (636 women and 495 men) aged 32–69 yr and 33–69 yr, respectively. In addition, we analyzed a sub-cohort of individuals aged ≥50 yr (975 women and 847 men).

### Assessment of physical activity

Data on leisure time physical activity was assessed by a self-administrated multiple-choice questionnaire that included several lifestyle and health-related questions including physical activity. A three-page questionnaire for the 2001 survey with two questions and a four-page questionnaire for the 2007–2008 survey with four questions concerning leisure time exercise and physical exertion accompanied the study invitation.

Subjects responded to the questions exploring the following topics: i. total level of physical activity, ii. exercise frequency, iii. exercise intensity and iv. exercise duration:

i. “Exercise and physical exertion in leisure time. If your activity varies much, for example between summer and winter, then give an average. The question refers only to the last twelve months”. Answers alternatives to this question were: 1) Reading, watching TV, or other sedentary activity?, 2) Walking, cycling, or other forms of exercise at least 4 h a week? (Including walking or cycling to place of work, Sunday-walking, etc.), 3) Participation in recreational sports, heavy gardening, etc.? (Note: duration of activity at least 4 h a week), 4) Participation in hard training or sports competitions, regularly several times a week.
  Based on their answers to this item, the subjects were divided into three groups: Sedentary (answer alternative 1), Moderately active (answer alternative 2), and Highly active (answer alternatives 3 and 4 combined).
ii. “How often do you exercise (i.e walking, skiing, swimming or training/sports)?” Answers alternatives to this question were: 1) Never, 2) Less than once a week, 3) Once a week, 4) 2–3 times a week, 5) Approximately every day
iii. “If you exercise - how hard do you exercise?” Answers alternatives to this question were: 1) Easy - you do not become short-winded or sweaty, 2) You become short-winded and sweaty, 3) Hard - you become exhausted
iv. “For how long time do you exercise? (give an average)” Answers alternatives to this question were: 1) Less than 15 min, 2) 15–29 min, 3) 30–60 min, 4) More than 1 h

The first question (i.) was common for both the 2001 and 2007–2008 surveys. All questions were multiple-choice questions and the participants were instructed to choose one of the response options. In addition, in order to study the association between change in physical activity from 2001 to 2007–2008 and vertebral fracture, the participants were grouped according to their physical activity level (questionnaire, question i.) in 2001 and 2007–2008. The following categories were created based on their changed or unchanged behavior: a) sedentary unchanged, b) reduced activity, c) increased activity and d) active unchanged [13].

### Ascertainment of vertebral fracture

Vertebral fracture assessment (VFA) was performed using vertebral morphometry, which is a quantitative method developed for identification of osteoporotic vertebral fractures based on the measurement of vertebral heights in dual-energy X-ray absorptiometry (DXA) scans. Although spine radiographs are generally considered to be the gold standard for the diagnosis of vertebral fractures [14, 15], the morphometric method is recognized for being easy, precise and using low radiation exposure [16–18]. When combined with BMD (bone mineral density) measurements, it is even argued it could become the “gold standard” [19].

VFA was performed in 2007–08 using DXA (Lunar Prodigy, GE Medical systems, Madison, WI, USA). Selection criteria (e.g. age) and random sampling were used to select participants eligible for DXA scans [12], and 3854 persons were invited to the DXA assessment, of which 2894 were randomly selected for VFA. After excluding blurred scans, 2886 VFA scans were further included. For this study, scans including 9–13 measured vertebras (Table 1) in the thoracic 4th to 12th vertebrae (T4-T12) and the lumbar 1st to 4th vertebrae (L1-L4) regions were included in data analysis. Participants with missing data on physical activity and confounders were excluded, leaving 1904 scans for the cross-sectional study and 1131 scans for the prospective study.

**Table 1:** Number of measured vertebras (*n* = 1904)

	No. measured vertebrae	*N*	Percent %
Women (*n* = 1030)	9	3	0.3
	10	2	0.2
	11	25	2.4
	12	88	8.5
	13	912	88.5
Men (*n* = 874)	9	2	0.2
	10	7	0.8
	11	52	5.9
	12	232	26.5
	13	581	66.5

All DXA scans were acquired according to a standard procedure set by GE Lunar Prodigy, Lunar Corp., Madison, USA, and in GE Lunar encore version 12.20. Daily calibration with phantom provided by the manufacturer was performed throughout the survey.

Specially trained technicians conducted the scanning according to the standardized protocol, and one of them performed the quality assessment of the total material afterwards. Determination of fracture types was done visually according to a standard set by GE Lunar Prodigy, i.e. grading of vertebral fracture type and severity were based on height measurements of the anterior, posterior and mid points of the vertebral bodies [20]. Fracture types were classified as wedge if the anterior height was the lowest, biconcave if the middle height was the lowest or crush if the posterior height was the lowest. Grade of the fracture severity was determined by percentagewise reduction in vertebral height in wedge, biconcave or crush vertebra. In this study, all types of fracture and mild to severe grade of the fracture with at least minimum of 20% reduction in vertebral body height were considered as a vertebral fracture.

For precision analysis of the VFA, a random sample of 50 participants was reanalyzed. The mean intra-class correlation coefficient was 0.82, 0.79, 0.82, and 0.84 for anterior, middle, posterior, and average height, respectively, all vertebrae considered. At the vertebrae with highest frequency of present deformity, exemplified by 7th and 12th thoracic vertebrae, the intra-class correlation coefficient varied between 0.77 and 0.92, with a mean of 0.86.

### Additional measurements

Left hip total bone mineral density (BMD; expressed as g/cm^2^) was measured using dual-energy X-ray absorptiometry (DXA) (Lunar Prodigy, GE Medical systems, Madison, WI, USA). Height and weight were measured at the physical examination in light clothing to nearest centimeter and half-kilogram respectively. Smoking (current/previous or never), having a diagnosis for osteoporosis (current/previous or no), osteoporosis medication (current/previous or no), and use of hormones (current or no) were self-reported.

### Statistical analyses

Two sets of analyses were performed. Firstly, associations between physical activity parameters (i.e. physical activity level, duration, intensity, and frequency) and vertebral fracture, available from the 2007–2008 survey, were analyzed using logistic regression. Subsequently, another set of logistic regression was performed to analyze associations between changes in physical activity from the 2001 survey to the 2007–2008 survey, and vertebral fracture. For each set of analyses, three models were performed:

1. An unadjusted model (only the physical activity variables included as predictors of vertebral fracture),
2. An age-adjusted model (age as a continuous variable in addition to physical activity), and
3. A multiple-adjusted model, in which additional adjustments were made for height, weight, left hip total BMD and self-reported smoking, osteoporosis, osteoporosis medication, and use of hormones in women only.

Total leisure time physical activity level was included in the models as a categorical variable to test differences in fracture risk between categories, and as a continuous variable in order to investigate linear trend across the categories (only in the cross-sectional analyses). All analyses were repeated for men and women separately, as well as in a sub-cohort of those individuals who reached ≥50 yr (women *n* = 975 and men *n* = 847) during the 2007–2008 survey.

## Results

### Sample characteristics

Sample characteristics for the participants from the 2007–2008 examination are shown in Table 2. The study includes 1030 women and 874 men aged 38–87 yr and 40–87 yr, respectively. Mean age was highest in the sedentary women, whereas men differed less than 0.4 years between physical activity categories. Sedentary women and men had the highest bodyweight. We observed similar prevalences of vertebral fracture in all physical activity groups in women, whereas in men the lowest prevalence of vertebral fracture was observed in the sedentary group. In total, 98 (9.5%) of all women and 112 (12.8%) of all men had vertebral fracture in 2007–2008. Moreover, the prevalence of women self-reporting an osteoporosis diagnosis was highest among the sedentary women.

**Table 2:** Sample characteristics in 2007–2008, stratified by level of physical activity

	Sedentary	Moderately active	Highly active
Women (*n* = 1030)	152	757	121
Age, years; M(SD)	64.6 (9.8)	63.2 (8.8)	61.2 (8.4)
Height, m; M(SD)	1.63 (6.81)	1.63 (6.29)	1.64 (6.96)
Weight, kg; M(SD)	74.0 (15.7)	70.5 (12.2)	68.2 (10.3)
Vertebral fracture (n; %)	15 (9.9)	71 (9.4)	12 (9.9)
Smoking daily, current or previous (n; %)	91 (59.9)	447 (59.0)	80 (66.1)
Osteoporosis, current or previous (n; %)	14 (9.2)	58 (7.7)	6 (5.0)
Osteoporosis medication, current or previous (n; %)	9 (5.9)	57 (7.5)	6 (5.0)
Current use of hormones^a^ (n; %)	11 (7.2)	57 (7.5)	9 (7.4)
Left hip total BMD, g/cm^2^; M(SD)	0.906 (0.134)	0.913 (0.131)	0.903 (0.118)
Men (*n* = 874)	142	518	214
Age, years; M(SD)	64.0 (10.0)	64.2 (9.1)	63.8 (8.9)
Height, m; M(SD)	1.76 (6.03)	1.76 (6.62)	1.76 (6.55)
Weight, kg; M(SD)	88.0 (12.8)	84.0 (11.8)	83.0 (11.6)
Vertebral fracture (n; %)	13 (9.2)	72 (13.9)	27 (12.6)
Smoking daily, current or previous (n; %)	105 (73.9)	388 (74.9)	133 (62.1)
Osteoporosis, current or previous (n; %)	2 (1.4)	6 (1.2)	2 (0.9)
Osteoporosis medication, current or previous (n; %)	0 (0.0)	10 (1.9)	2 (0.9)
Left hip total BMD, g/cm^2^; M(SD)	1.030 (0.156)	1.025 (0.137)	1.044 (0.139)

Values are means (M) (standard deviation [SD]) or *n* (percentages)

^a^Only women

Sample characteristics for the prospective follow-up study of 1131 subjects in 2001 are shown in Table 3. In both men and women, the sedentary individuals were approximately 2 years younger than moderately and highly active subjects. The highest mean weight also was found in the sedentary group, whereas height did not vary substantially between the groups.

**Table 3:** Sample characteristics in 2001, stratified by level of physical activity

	Sedentary	Moderately active	Highly active
Women (*n* = 636)	102	474	102
Age, years; M(SD)	56.7 (7.0)	58.3 (7.6)	58.1 (9.5)
Height, cm; M(SD)	162.9 (6.4)	163.3 (6.2)	163.0 (6.9)
Weight, kg; M(SD)	74.4 (14.5)	70.2 (11.8)	68.9 (12.5)
Smoking daily, current or previous (n; %)	66 (64.1)	279 (58.9)	38 (63.3)
Osteoporosis, current or previous (n; %)	2 (2.0)	17 (3.6)	3 (5.0)
Osteoporosis medication, current or previous (n; %)	3 (2.9)	19 (4.0)	2 (3.3)
Men (*n* = 495)	82	319	94
Age, years; M(SD)	57.2 (9.0)	59.9 (7.7)	59.0 (7.9)
Height, cm; M(SD)	176.8 (6.2)	175.8 (6.4)	177.0 (5.4)
Weight, kg; M(SD)	88.0 (12.8)	83.7 (11.6)	83.4 (11.2)
Smoking daily, current or previous (n; %)	57 (69.5)	243 (76.2)	67 (71.3)
Osteoporosis, current or previous (n; %)	1 (1.2)	2 (0.6)	1 (1.1)
Osteoporosis medication, current or previous (n; %)	0 (0.0)	3 (0.9)	1 (1.1)

Values are means (M) (standard deviation [SD]) or *n* (percentages)

^a^Only women

**Fig. 1:**
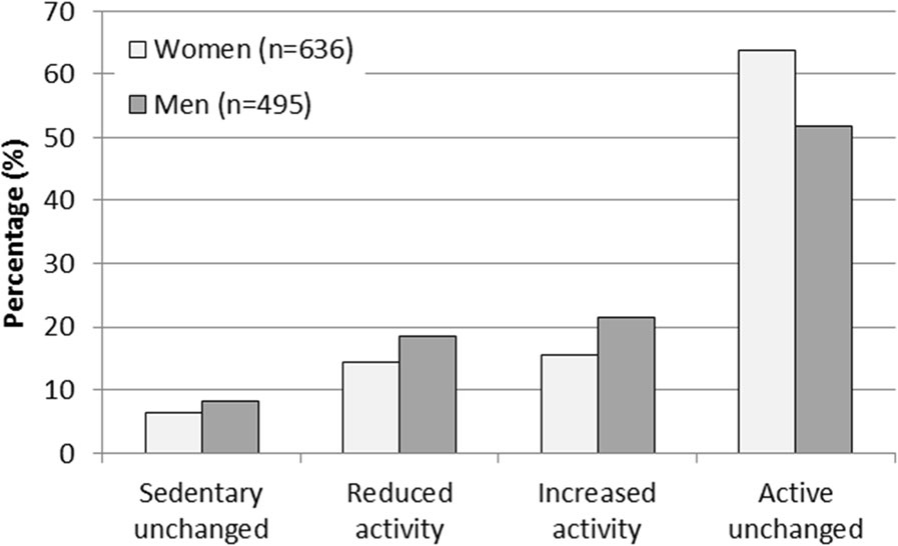
Change in physical activity level from 2001 to 2007–2008

Figure 1 presents changes in physical activity from 2001 to the follow-up in 2007–2008. The proportion of women who remained physically active was 63.8% and 6.3% remained sedentary, whereas 14.3% of the women reduced and 15.6% increased their activity levels. In men, 51.9% remained physically active and 8.1% remained sedentary, while 18.6% reduced and 21.4% increased their physical activity level.

### Physical activity and vertebral fractures

Table 4 presents the unadjusted, the age-adjusted and the multiple adjusted odds ratios (OR) with 95% confidence interval (CI) for vertebral fracture in relation to physical activity level, −duration, −intensity and -frequency. We found a significant association between physical activity intensity and vertebral fracture in the unadjusted model, with women in the “moderately/ high intensity” group being less likely to have a vertebral fracture as compared with women in the “low intensity” group (OR 0.53, 95% CI: 0.34–0.83). However, the association was no longer significant in the adjusted models. We found no further associations between any of the physical activity parameters and vertebral fracture in unadjusted, age-adjusted and multiple adjusted analyses. This applied to categorical physical activity, trends across categories, duration, intensity and frequency of the physical activity in both women and men. Analysis of subjects ≥50 yr (women *n* = 975 and men *n* = 847) gave similar results as the main analyses.

**Table 4:** Cross-sectional associations between leisure time exercise and physical exertion, and vertebral fracture

Exercise and physical exertion	Vertebral fractures (n)	Unadjusted OR	95% CI	Adjusted for age OR	95% CI	Multiple adjusted OR^a^	95% CI
Female (*n* = 1030)							
Physical activity level							
Low	15	Reference		Reference		Reference	
Moderate	71	0.95	0.53–1.70	1.09	0.60–1.99	1.13	0.59–2.13
High	12	1.01	0.45–2.24	1.36	0.60–3.10	1.44	0.61–3.42
Physical activity: linear trend		1.00	0.67–1.50	1.16	0.76–1.76	1.20	0.77–1.85
Duration							
0–29 min.	22	Reference		Reference		Reference	
30–60 min.	57	0.69	0.41–1.16	0.76	0.45–1.30	0.79	0.45–1.38
> 60 min.	19	0.71	0.37–1.37	0.78	0.41–1.52	0.78	0.39–1.58
Intensity							
Low	68	Reference					
Mod./high	30	0.53	0.34–0.83	0.75	0.47–1.21	0.82	0.50–1.35
Frequency							
< Once a week	8	Reference		Reference		Reference	
Once a week	20	1.24	0.53–2.91	1.05	0.44–2.51	1.17	0.47–2.90
2–3 times/week	48	1.27	0.58–2.76	1.08	0.49–2.39	1.07	0.47–2.43
Every day	22	1.22	0.52–2.84	1.03	0.43–2.42	1.01	0.41–2.49
Age (years)				1.08	1.05–1.10	1.04	1.00–1.07
BMD left hip (100 g/cm^2^)						0.97	0.95–0.99
Osteoporosis current/previous						3.94	1.54–10.09
Male (*n* = 874)							
Physical activity level							
Low	13	Reference		Reference		Reference	
Moderate	72	1.60	0.86–2.99	1.62	0.87–3.03	1.74	0.91–3.35
High	27	1.43	0.71–2.88	1.47	0.73–2.98	1.64	0.78–3.41
Physical activity: linear trend		1.13	0.83–1.55	1.15	0.84–1.58	1.20	0.86–1.67
Duration							
0–29 min.	23	Reference		Reference		Reference	
30–60 min.	63	0.94	0.56-1.57	1.00	0.60–1.68	1.09	0.63–1.88
> 60 min.	26	0.80	0.44–1.46	0.86	0.47–1.58	0.91	0.49–1.72
Intensity							
Low	51	Reference		Reference		Reference	
Mod./high	61	1.07	0.72–1.60	1.27	0.84–1.92	1.38	0.90–2.12
Frequency							
< Once a week	15	Reference		Reference		Reference	
Once a week	30	1.44	0.75-2.79	1.41	0.73–2.75	1.31	0.66–2.59
2–3 times/week	46	1.28	0.69-2.37	1.19	0.64–2.21	1.24	0.66–2.35
Every day	21	1.42	0.70–2.88	1.16	0.56–2.38	1.24	0.59–2.60
Age (years)				1.04	1.02–1.07	1.04	1.01–1.06
BMD left hip (100 g/cm^2^)						0.96	0.95–0.98
Osteoporosis current/previous						4.44	0.73–27.07

^a^Adjusted for age, height, weight, smoking, osteoporosis, osteoporosis medication, left hip total BMD, and use of hormones (women only)

Table 5 presents results for the change in physical activity as a risk factor for vertebral fracture as unadjusted, age-adjusted and multiple adjusted odds ratios (OR) with 95% confidence interval (CI). We found no associations between change in leisure time exercise and physical exertion, and risk of vertebral fracture in unadjusted, age-adjusted and multiple adjusted analyses. This applied to change in physical activity levels in categories, trends across categories, in both women and men.

**Table 5:** Associations between change in physical activity levels and vertebral fracture

Exercise and physical exertion	Vertebral fractures (n)	Unadjusted OR	95% CI	Adjusted for age OR	95% CI	Multiple adjusted OR^a^	95% CI
Female (*n* = 636)							
Physical activity level							
Sed. unchanged	3	Reference		Reference		Reference	
Reduced activity	6	0.87	0.21–3.67	0.79	0.18–3.34	0.81	0.18–3.62
Increased activity	8	1.08	0.27–4.31	1.18	0.29–4.71	1.24	0.29–5.26
Active unchanged	47	1.62	0.48–5.44	1.56	0.46–5.28	1.54	0.43–5.50
Age (years)				1.07	1.03–1.12	1.05	1.00–1.10
BMD left hip (100 g/cm^2^)						0.97	0.94–0.99
Osteoporosis current/previous						3.61	1.06–12.31
Male (*n* = 495)							
Physical activity level							
Sed. unchanged	4	Reference		Reference		Reference	
Reduced activity	14	1.62	0.50–5.25	1.58	0.48–5.18	2.05	0.57–7.42
Increased activity	17	1.72	0.54–5.46	1.75	0.55–5.61	2.23	0.63–7.87
Active unchanged	36	1.47	0.49–4.37	1.38	0.46–4.13	1.81	0.54–6.02
Age (years)				1.05	1.01–1.09	1.04	1.00–1.08
BMD left hip (100 g/cm^2^)						0.97	0.94–0.99
Osteoporosis current/previous						14.09	1.13–176.20

^a^Adjusted for age, height, weight, smoking, osteoporosis, osteoporosis medication, left hip total BMD, and use of hormones (women only)

## Discussion

With this population-based study, we wanted to fill a research gap in the association of physical activity and vertebral fracture. Only a few studies have shown interest in this topic, although vertebral fracture is a major cause for morbidity and reduced quality of life. Therefore, preventing vertebral fractures would be beneficial for the individual, but also for the society in terms of reduced economic burden. In this study, we did not find any statistically or clinically significant associations between levels of leisure time physical activity and prevalent vertebral fracture. Moreover, no association between exercise intensity, frequency or duration, and vertebral fracture was found. In subjects with prospective data, we did not find any significant associations between 6 year changes in leisure time physical activity and risk of vertebral fracture. Furthermore, in a sub-cohort of individuals aged ≥50 yr, no significant associations between physical activity and prevalent vertebral fracture were found.

Comparison with other studies is challenging because of the different methods of assessing physical activity and technologies used to identify vertebral fracture. A few cross-sectional studies [21, 22] have investigated associations between physical activity and vertebral fracture, although these studies focused on occupational physical activity and other lifestyle factors, rather than on recreational sports or physical activity. Ling et al. [22] studied Chinese women aged ≥50 and found that women whose work had involved heavy physical labor had a lower prevalence of vertebral fracture than women who had more sedentary jobs. Kwok et al. [21] studied ethnic Asian men and women aged ≥65, and found that farmer occupation was significantly associated with increased risk for the vertebral fracture in women. The differences in findings between the present study and these previous studies could be explained by ethnicity and differences in age and definition of physical activity. Moreover, it is reasonable to assume that farming labor could be rather wearing in its duration and as an activity itself. Previous studies have shown a steady increase in sedentary occupational activity over the last decades, whereas leisure time physical activity increased [23]. Such changes in occupational physical activity make the comparison between different studies difficult. Waterloo et al. [24] studied a larger cohort from the 2007–2008 Tromsø Study (*n* = 2887) to identify risk factors for vertebral fracture, including high vs. low physical activity, showing results that support the present study by concluding that physical activity was not associated with vertebral fracture. Similar to our study, other prospective observational studies [25–27] in Europe did not find significant associations between physical activity and vertebral fracture.

Moreover, our findings are partly consistent with a prospective cohort study with 3.7-year follow-up time on vertebral fracture in North American women 65 years of age or older [28]. They did not find any significant associations between total physical activity and vertebral fracture, although high physical activity intensity was associated with a reduced risk of vertebral fracture. In our study, a significant association between physical activity intensity and reduced risk of vertebral fracture in women was found only in the unadjusted model, but this association was no longer significant when adjusting for multiple confounders. In our opinion, this indicates that the relationship of physical activity intensity with reduced risk of vertebral fracture is complex and possibly confounded and mediated by other factors. Age, in particular, appears to mediate the relationship. Ageing is associated with decreasing physical activity levels [23]. As seen in our study, the significant unadjusted relationship between physical activity and risk of vertebral fractures was no longer significant when adjusting for age and other possible confounders.

Risk factors such as advancing age have been found to be associated with vertebral fracture [22, 29, 30], and older age was also significantly associated with vertebral fracture in our study, both in women and in men. Still, physical activity was not associated with vertebral fracture in our sub-cohort of men and women aged ≥50 yr. The main cohort was not substantially larger than the sub-cohort, only 55 women and 27 men less than in the main cohort, which might have led to very similar outcome in both cohorts.

A large multinational multicenter case-control study [31] examining men and women aged 50–79 yr concluded that walking/cycling ≥30 min/day was associated with reduced risk of vertebral fracture in middle-aged and elderly women, while heavy physical activity in early and middle adulthood was associated with increased risk of vertebral fracture in men. However, case-control studies are vulnerable to bias, such as recall bias: the outcome has already occurred, and cases and controls might recall the exposure differentially based on the knowledge about the outcome. Furthermore, selection bias might occur when controls are included or excluded based on their characteristics, and this may affect the outcome.

In all, the findings concerning associations between physical activity and vertebral fracture are inconsistent, probably partly due to different assessment methods, ethnicity, sex and age used in the different studies. However, studies of physical activity as rehabilitation regime in patients with vertebral fracture show that physical activity may reduce pain and improve quality of life, suggesting that physical activity may be of particular importance after a vertebral fracture has occurred [32], in addition to many benefits for health in general [33].

Results from this study should be viewed in light of possible limitations. Although X-ray technology is considered gold standard in vertebral morphometry, we used DXA scans without x-ray quality in our study. DXA scans are reported to be more accurate in measuring moderate and severe than mild vertebral fracture [34], which can lead to underestimation of the vertebral fractures. The study would have benefited from longitudinal vertebral fracture data, which is currently not available in the Tromsø study. Vertebral fractures often result from osteoporosis and can develop over time [35]. No DXA scans for vertebral fracture assessment were taken in 2001. Therefore, we cannot state the exact timing of the fracture, only that it has occurred before the 2007–2008 Tromsø Study DXA scan measurements. Furthermore, vertebral fractures can cause reduced quality of life due to pain, reduced physical function and sleeping disorders [1]. The change in physical activity levels in this study might therefore have occurred due to vertebral fracture. Assessing physical activity with self-administrated multiple-choice questionnaire can cause recall bias. Nevertheless, Emaus et al. [36] studied total level of self-reported physical activity assessed by the Tromsø Study questionnaire and objectively measured physical activity assessed by Actigraph activity monitor in 313 healthy men and women drawn from the Tromsø Study population (2007–2008), concluding that the questionnaire had acceptable validity. Kurtze et al. [37] performed a validation study on 108 healthy men using the same questions on exercise frequency, exercise intensity and exercise duration as in our study. They concluded that the questionnaire was appropriate for assessing physical activity in epidemiological studies.

No objective physical activity data was accessible from the Tromsø study in 2001 and 2007–2008. Moreover, we do not know the type of physical activity, only volume and intensity. Activities causing forces directed on the vertebrae and positions of the vertebrae during certain types of physical activity might cause increased risk of vertebral fracture [38]. In addition, for example running and weight bearing exercise is seen as beneficial for bone health and preventing fractures [7], whereas cycling seems not to contribute as much to the osteogenic stimulus that is needed for improving bone health [39]. Therefore, being able to identify the type of physical activity, would help us to gain more detailed knowledge on associations between physical activity and vertebral fracture. Also, our analysis did not include additional confounders such as dietary factors, falls at baseline, or general health status.

## Conclusions

Our findings suggest that physical activity, which is an important health promoting factor, does not play any major role in preventing vertebral fracture in adult and elderly women and men. Future studies on this topic might benefit from objectively measured physical activity and a longitudinal study design in predicting vertebral fracture.

## Data Availability

According to the data extradition contract, the extracted data file cannot be shared publicly due to ethical and privacy concerns. However, raw data are available upon application via the web portal http://tromsoundersokelsen.uit.no/tromso/.

## Abbreviations

BMD: Bone mineral density
CI: Confidence interval
DXA: Dual-energy X-ray absorp­tiometry scans
OR: Odds ratio
VFA: Vertebral fracture assessment

## References

[1] Lips P, van Schoor NM (2005). Quality of life in patients with osteoporosis. Osteoporos Int.

[2] Hasserius R, Karlsson MK, Jonsson B, Redlund-Johnell I, Johnell O (2005). Long-term morbidity and mortality after a clinically diagnosed vertebral fracture in the elderly--a 12- and 22-year follow-up of 257 patients. Calcif Tissue Int.

[3] Cosman F, de Beur SJ, LeBoff MS, Lewiecki EM, Tanner B, Randall S (2014). Clinician's guide to prevention and treatment of osteoporosis. Osteoporos Int.

[4] Ballane G, Cauley JA, Luckey MM, El-Hajj Fuleihan G (2017). Worldwide prevalence and incidence of osteoporotic vertebral fractures. Osteoporos Int.

[5] Waterloo S, Ahmed LA, Center JR, Eisman JA, Morseth B, Nguyen ND (2012). Prevalence of vertebral fractures in women and men in the population-based Tromso study. BMC Musculoskelet Disord.

[6] Hernlund E, Svedbom A, Ivergard M, Compston J, Cooper C, Stenmark J (2013). Osteoporosis in the European Union: medical management, epidemiology and economic burden. A report prepared in collaboration with the international Osteoporosis Foundation (IOF) and the European Federation of Pharmaceutical Industry Associations (EFPIA). Arch Osteoporos.

[7] Kohrt WM, Bloomfield SA, Little KD, Nelson ME, Yingling VR (2004). American College of Sports M. American College of Sports Medicine position stand: physical activity and bone health. Med Sci Sports Exerc.

[8] Kemmler W, Haberle L, von Stengel S (2013). Effects of exercise on fracture reduction in older adults: a systematic review and meta-analysis. Osteoporos Int.

[9] Giangregorio LM, Papaioannou A, Macintyre NJ, Ashe MC, Heinonen A, Shipp K (2014). Too fit to fracture: exercise recommendations for individuals with osteoporosis or osteoporotic vertebral fracture. Osteoporos Int.

[10] Olsen CF, Bergland A (2014). The effect of exercise and education on fear of falling in elderly women with osteoporosis and a history of vertebral fracture: results of a randomized controlled trial. Osteoporos Int.

[11] Giangregorio LM, Macintyre NJ, Thabane L, Skidmore CJ, Papaioannou A. Exercise for improving outcomes after osteoporotic vertebral fracture. Cochrane Database Syst Rev. 2013;(1):CD008618. 10.1002/14651858.CD008618.pub2 PMC5104540 23440829

[12] Jacobsen BK, Eggen AE, Mathiesen EB, Wilsgaard T, Njolstad I (2012). Cohort profile: the Tromso study. Int J Epidemiol.

[13] Morseth B, Emaus N, Wilsgaard T, Jacobsen BK, Jorgensen L (2010). Leisure time physical activity in adulthood is positively associated with bone mineral density 22 years later. The Tromso study. Eur J Epidemiol.

[14] Middleton ET, Gardiner ED, Steel SA (2009). Which women should be selected for vertebral fracture assessment? Comparing different methods of targeting VFA. Calcif Tissue Int.

[15] Diacinti D, Guglielmi G (2010). Vertebral morphometry. Radiol Clin N Am.

[16] El Maghraoui A, Roux C (2008). DXA scanning in clinical practice. QJM..

[17] Grigoryan M, Guermazi A, Roemer FW, Delmas PD, Genant HK (2003). Recognizing and reporting osteoporotic vertebral fractures. Eur Spine J.

[18] Ensrud KE, Schousboe JT (2011). Clinical practice. Vertebral fractures. N Engl J Med.

[19] Sanfelix-Genoves J, Reig-Molla B, Sanfelix-Gimeno G, Peiro S, Graells-Ferrer M, Vega-Martinez M (2010). The population-based prevalence of osteoporotic vertebral fracture and densitometric osteoporosis in postmenopausal women over 50 in Valencia, Spain (the FRAVO study). Bone.

[20] Kim N, Rowe BH, Raymond G, Jen H, Colman I, Jackson SA (2004). Underreporting of vertebral fractures on routine chest radiography. AJR Am J Roentgenol.

[21] Kwok AW, Leung JC, Chan AY, Au BS, Lau EM, Yurianto H (2012). Prevalence of vertebral fracture in Asian men and women: comparison between Hong Kong, Thailand, Indonesia and Japan. Public Health.

[22] Ling X, Cummings SR, Mingwei Q, Xihe Z, Xioashu C, Nevitt M (2000). Vertebral fractures in Beijing, China: the Beijing osteoporosis project. J Bone Miner Res.

[23] Morseth B, Jacobsen BK, Emaus N, Wilsgaard T, Jorgensen L (2016). Secular trends and correlates of physical activity: the Tromso study 1979-2008. BMC Public Health.

[24] Waterloo S, Nguyen T, Ahmed LA, Center JR, Morseth B, Nguyen ND (2012). Important risk factors and attributable risk of vertebral fractures in the population-based Tromso study. BMC Musculoskelet Disord.

[25] Finigan J, Greenfield DM, Blumsohn A, Hannon RA, Peel NF, Jiang G (2008). Risk factors for vertebral and nonvertebral fracture over 10 years: a population-based study in women. J Bone Miner Res.

[26] Roy DK, O'Neill TW, Finn JD, Lunt M, Silman AJ, Felsenberg D (2003). Determinants of incident vertebral fracture in men and women: results from the European prospective osteoporosis study (EPOS). Osteoporos Int.

[27] Kerschan-Schindl K, Uher E, Kainberger F, Kaider A, Ghanem AH, Preisinger E (2000). Long-term home exercise program: effect in women at high risk of fracture. Arch Phys Med Rehabil.

[28] Gregg EW, Cauley JA, Seeley DG, Ensrud KE, Bauer DC, SoOFR G (1998). Physical activity and osteoporotic fracture risk in older women. Ann Intern Med.

[29] Cooper C, O'Neill T, Silman A (1993). The epidemiology of vertebral fractures. European vertebral osteoporosis study group. Bone.

[30] Yakemchuk V, Beaumont LF, Webber CE, Gulenchyn KY, Jager PL (2012). Vertebral fracture prevalence in a referral population of 750 Canadian men and women. Clin Radiol.

[31] Silman AJ, ONeill TW, Cooper C, Kanis J, Felsenberg D (1997). Influence of physical activity on vertebral deformity in men and women: results from the European vertebral osteoporosis study. J Bone Miner Res.

[32] Giangregorio LM, MacIntyre NJ, Heinonen A, Cheung AM, Wark JD, Shipp K (2014). Too fit to fracture: a consensus on future research priorities in osteoporosis and exercise. Osteoporos Int.

[33] Warburton DE, Nicol CW, Bredin SS (2006). Health benefits of physical activity: the evidence. CMAJ.

[34] Middleton ET, Steel SA (2008). Routine versus targeted vertebral fracture assessment for the detection of vertebral fractures. Osteoporos Int.

[35] Delmas PD, van de Langerijt L, Watts NB, Eastell R, Genant H, Grauer A (2005). Underdiagnosis of vertebral fractures is a worldwide problem: the IMPACT study. J Bone Miner Res.

[36] Emaus A, Degerstrom J, Wilsgaard T, Hansen BH, Dieli-Conwright CM, Furberg AS (2010). Does a variation in self-reported physical activity reflect variation in objectively measured physical activity, resting heart rate, and physical fitness? Results from the Tromso study. Scand J Public Health.

[37] Kurtze N, Rangul V, Hustvedt BE, Flanders WD (2008). Reliability and validity of self-reported physical activity in the Nord-Trondelag health study: HUNT 1. Scand J Public Health.

[38] Giangregorio L, El-Kotob R (2017). Exercise, muscle, and the applied load-bone strength balance. Osteoporos Int.

[39] Olmedillas H, Gonzalez-Aguero A, Moreno LA, Casajus JA, Vicente-Rodriguez G (2012). Cycling and bone health: a systematic review. BMC Med.

